# Association between the Internet Gaming Disorder and Anxiety and Depression among University Students during COVID-19 Pandemic

**DOI:** 10.3390/healthcare11081103

**Published:** 2023-04-12

**Authors:** Mohd Fariz Idris, Suriati Mohamed Saini, Shalisah Sharip, Nur Farahaizan Idris, Nur Fadilah Ab Aziz

**Affiliations:** 1 Department of Psychiatry, Faculty of Medicine, University Kebangsaan Malaysia, Jalan Yaacob, Kuala Lumpur 56000, Malaysia; 2 Department of Psychiatry, Hospital Tengku Ampuan Rahimah, Jalan Langat, Klang 41200, Malaysia; 3 Polytechnic Sultan Salahuddin Abdul Aziz Shah, Persiaran Usahawan, Shah Alam 40150, Malaysia; 4 Faculty of Engineering, Tenaga Nasional University, Jalan Ikram-Uniten, Kajang 43000, Malaysia

**Keywords:** Internet Gaming Disorder, IGD, depression, anxiety, stress, associated factors, IGD9-SF, DASS-21, prevalence rate

## Abstract

**Introduction**: Internet gaming is now a major concern since its overuse has had a detrimental impact on people’s well-being. This study aims to investigate the association between Internet Gaming Disorder and depression, anxiety, and stress, as well as gaming elements during the COVID-19 pandemic, among university students. **Methods:** The cross-sectional study involved 213 students from two different institutions who were randomly selected. The participants were required to complete three sets of online questionnaires via Google Forms. The online questionnaire consists of the Internet Gaming Disorder Scale-Short Form (IGD9-SF) and the Depression, Anxiety, and Stress Scale (DASS-21). **Results:** The prevalence rate of IGD among university students during the COVID-19 pandemic was 9.86%. Bivariate analysis revealed biological sex (*p*-value = 0.011), preferred gaming platforms (*p*-value = <0.001), game gameplay (*p*-value = 0.03), history of substance use (*p*-value = <0.001), and stress (*p*-value = <0.001) to be associated with IGD. Meanwhile, binary logistic regression demonstrated that males have a higher risk of developing IGD compared with females (adjusted odds ratio (AOR) = 3.426, *p*-value 0.015, CI = 1.27–9.21). Students who used consoles as their preferred gaming platform were 13 times more likely to develop IGD in comparison to another platform (AOR = 13.031, *p*-value = 0.010, 95% CI = 1.87–91.02). Extensive gaming duration of more than 4 h a day showed a higher risk of developing IGD (AOR = 8.929, *p*-value 0.011, CI = 1.659–48.050). High-stress levels significantly increased the risk of IGD (AOR = 13.729, *p*-value = 0.001, 95% CI = 2.81–67.1). **Conclusion:** The prevalence of IGD among university students was high during the COVID-19 pandemic. Thus, interventions for reducing stress among university students should be implemented to reduce the risk of IGD.

## 1. Introduction

Gaming has rapidly emerged since the last decade, and most countries nowadays have recognised gaming as one major branch of competitive sport or e-sport [1,2,3]. The emergence of internet gaming has raised concern since there seems to be an overuse of internet gaming among the public [4]. WHO (World Health Organisation) has proposed and introduced a diagnosis of “Gaming Disorder” in their International Classification of Diseases 11th Revision (ICD-11) to justify their concern about the negative impact of gaming on the public [5]. In 2013, the Internet Gaming Disorder (IGD) was included as one of the conditions under further study in the Diagnostic and Statistical Manual of Mental Disorders (DSM-V) [6].

The prevalence of IGD ranges from 0.7% to 25.5% worldwide, with the Asian population showing a higher rate of IGD compared with the North American and European populations [7]. IGD prevalence is higher among males than females and more common among the young generation [5]. In Malaysia, the popularity of e-sports started to rise in the last ten years, when more e-sports clubs were established in higher educational institutions. A survey study showed that mobile online gaming in Malaysia alone involves nearly 14 million people, with a ratio of both men and women of 55.3% and 44.7%. Of those numbers, 39.5% are aged between 25 and 34 years, and about 23.8% are younger players aged between 18 and 24 years old. Over the years, many studies have focused on the factors associated with IGD. Social-related issues such as bullying and a history of physical and verbal assault are among the precipitating factors that led to IGD [8,9]. Insufficient social support, which includes family and peer support and interpersonal difficulties associated with poor social skills, contributed to an increased risk of internet addiction and IGD [10,11].

Despite being accepted by the public, online gaming has negative repercussions. Incidents such as cyberbullying, hate crimes, the use of inappropriate words, and sexual harassment have been recurrent issues mainly among players of massively multiplayer online games (MMOGs) [8]. Another concerning issue is inappropriate content that promotes inappropriate acts. The most common content involved violent conduct, such as the use of firearms or massacres, sexual misconduct, and pornography. The overuse of online gaming caused a substantial increase in the amount of playing time among gamers, hence leading to other issues such as sleep deprivation, physical illness, as well as an unhealthy diet and lifestyle [12]. DiFrancisco-Donoghue, Balentine, Schmidt, and Zwibel [13] conducted a study exploring health-related issues among gamers and discovered that players who play between 3 and 10 h per day might significantly compromise their physical well-being. A local study by Rasdi and Rusli [14] discovered how prolonged screen time leads to poor academic performance and reduced productivity. In extreme cases, intense gaming activity also leads to agoraphobia, social withdrawal, irritability, depression, decreased self-esteem, and sometimes refusal to go to work or school, leading to impairment in multiple areas of life [15].

Anxiety, depression, and Attention Deficit Hyperactive Disorder (ADHD) were observed as strongly associated with IGD [16,17,18,19,20]. Mihara and Higuchi [7] discussed how comorbid psychiatric illnesses act as both risk factors and consequences that can lead to IGD. In addition, a study reported that individuals with IGD had an increased tendency to develop worse depressive symptoms and to experience depressive symptoms during the remission from IGD [21]. In a different study, individuals with IGD presented a decreased level of resilience, higher perceived stress, and higher levels of depression [22]. At the same time, IGD showed a bi-directional relationship with anxiety disorder [23]. A correlational study had shown that IGD subjects with Generalised Anxiety Disorder (GAD) have higher depressive and anxiety scores than those without IGD, besides being eight times more likely to develop IGD [24]. Psychological stress occurs due to significant life events and is often associated with other psychiatric illnesses, including IGD, as individuals with IGD tend to deal with more stress and are less resilient [22,25]. Other vital factors, such as poor psychosocial support and interpersonal factors, appear to play a significant role in the increased risk of developing IGD [18,19,26].

During the COVID-19 pandemic, Malaysia was one of the countries that was significantly affected [27]. The Malaysian government had imposed a Movement Control Order (MCO) in March 2020 to cope with the situation, which resulted in the closure of many economic and academic activities, travel bans, as well as movement restrictions within the states. The concern about contracting the COVID-19 illness and its impact has led to significant psychological stress and other mental illnesses. To cope with the lockdown restriction and other psychological issues that were on the rise following MCO, many individuals began using gaming activities as escapism, which resulted in IGD. In addition, implementing MCO has forced schools and universities to close. The government then introduced home-based learning and teaching, whereby students and teaching staff were required to utilise computers and devices as a communication medium. Nevertheless, with the increased amount of unoccupied time and exposure to the device, it has also resulted in an increased amount of time spent playing games that led to IGD [28].

Several studies have explored the association between IGD and other primary psychiatric illnesses and psychological stress. However, only a few of them were conducted during the pandemic, when the nation faced a national health crisis. Therefore, this study aimed to investigate the association between Internet Gaming Disorder and depression, anxiety, and stress, as well as gaming elements during the COVID-19 pandemic.

## 2. Material and Methods

### 2.1. Design and Procedure

This study was a cross-sectional study involving all levels of students at two different higher education institution campuses in the Klang Valley, which were Polytechnic Sultan Salahuddin Abdul Aziz Shah in Shah Alam and University Tenaga Nasional (UNITEN) in Kajang. Approval from the University Kebangsaan Malaysia (UKM) ethics committee was initially obtained before data collection (JEP-2021-901). All participants had to fulfil the inclusion criteria of being between 18 and 40 years old, with an adequate understanding of English and Malay, and being able to provide informed consent before study entry. Participants who were previously diagnosed with ADHD, autism, or other developmental disorders and were unable to understand or speak the language used in this study (English or Malay) or who could not give their consent to participate in this study, were excluded from this study. All participants were provided with an information sheet explaining this study’s aims and purposes. The confidentiality of participants’ details and submitted data was assured.

The sample size was calculated based on a prevalence rate of 17.7% [29]. The degree of accuracy for the study was set at 0.05. Based on the calculation, the minimal sample size required was 186. However, considering the possibility of any subject dropout, this study aimed to recruit an additional 20% of the samples, thus increasing the number of study subjects to 222. A total of 213 participants who met the inclusion criteria were recruited through a random sampling process using a multistage cluster sampling technique. All participants were given a set of self-rated and validated questionnaires containing five different parts, including participants’ demographic background, internet gaming, and affective state. The questionnaires were distributed via participants’ e-mail and registered in the institution’s data system in Google Form.

The sociodemographic questionnaire is a self-reported questionnaire that comprises information on the participants’ age, ethnicity, sex, preferred gaming platform, duration of gaming, and history of substance use/addiction. The Internet Gaming Disorder Short Form 9 Test (IGD SF-9 Test) Malay language version is a brief, standardised psychometric tool used to assess Internet Gaming Disorder (IGD) based on the criteria as suggested by the American Psychiatric Association in the latest edition of the Diagnostic and Statistical Manual of Mental Disorders (DSM-5) [6,30]. The IGDS9-SF is a tool that contains a total of nine items reflecting all nine criteria for IGD as per the DSM-5. Each item was rated from one point for never to five points for very often. Participants with the disorder can be differentiated if they score more than 36 points out of a maximum of 45 points. IGDS9-SF has a sensitivity and specificity of 98.0% and 91.9%, respectively, with a Cronbach alpha of 0.96 for the English version and 0.87 for the validated Malay version [30,31]. The Depression, Anxiety, and Stress Scale (DASS) has been widely used to screen for depression, anxiety, and stress among adults and adolescents within the community, besides having good psychometric properties. DASS-21 is a self-reported scale that consists of 21 short items, whereas the score of the scale is divided into three subscales (anxiety, depression, and stress), with each of the subscales containing seven items. Each was rated from 0, meaning that the items stated did not apply to the responder at all, to a maximum score of 3, meaning that the items stated did apply to the responder most of the time. The total score of each subscale was calculated and then classified into normal, mild, moderate, severe, and extreme severe to indicate the severity of the symptom. This study utilised 21 items from the English version and the validated Bahasa Malaysia version of the DASS-21. The DASS-21 Malay language version has an internal consistency of 0.84, 0.74, and 0.89 for the depression, anxiety, and stress sections, respectively [32]. The DASS-21 English version was identical to Cronbach’s alpha, with 0.81, 0.73, and 0.81 for each similar section [33]. Data collection was carried out during the third wave of the Movement Control Order (MCO) period between 1 May 2022 and 30 June 2022, a few weeks before the Malaysian government lifted the MCO.

### 2.2. Data Analysis

Data were recorded and analysed using SPSS version 27. A descriptive analysis was performed to determine the prevalence of IGD as well as the sociodemographic characteristics, preferred gaming platforms, duration of gaming, and history of substance use/addiction in the population of this study. The results were presented as frequency and percentage for categorical data and mean (standard deviation) for continuous data. A bivariate analysis was conducted using the Chi-square test and Student *t*-test to determine the association between all independent variables and IGD. Binary logistic regression (backward LR) was performed on significant independent variables in the bivariate analysis to determine the predictors of IGD. By using the Chi-square test, the statistical significance of the relationship between categorical variables involved in the study can be determined. Meanwhile, binary logistic regression helps to estimate the degree of relationship between the dependent variable (IGD) and independent variables (sociodemographic, anxiety, depression, and stress). All *p*-values were set to less than 0.05.

## 3. Result

This study involved 213 participants (Table 1). Most participants were male (54.9%). In terms of ethnicity, the Malay population was the highest, followed by Indian, Chinese, and others. Most participants were young adults aged between 18 and 28 years old, with a mean age of 21.37. More than 70% of participants use a handphone or tablet as a medium to play games. During the pandemic period, most participants showed an increase in playing time compared with the prior pandemic (91.5%). Only 7% of the total participants were involved in illegal substance use.

Table 2 illustrates the association between sociodemographic characteristics, gaming profile including platform and duration, substance usage, depression, anxiety, and stress with IGD (*n* = 213). Sex (*χ^2^* = 6.538, df = 1, *p*-value = 0.011), preferred gaming platform (χ^2^ = 14.478, df = 2, *p*-value < 0.001), duration of playing time (χ^2^ = 11.813, df = 2, *p*-value = 0.03), history of substances (χ^2^ = 16.495, df = 1, *p*-value < 0.001), anxiety (χ^2^ = 5.192, df = 1, *p*-value = 0.023), and stress (χ^2^ = 15.445, df = 1, *p*-value < 0.001) showed a significant association with IGD. 

Table 3 shows the binary logistic regression between the independent variables with IGD. The table contained statistically significant independent variables where males have a higher risk of developing IGD than females (adjusted odds ratio (AOR) = 3.426, *p*-value 0.015, CI = 1.27–9.21). Additionally, the odds of having IGD were 13 times more likely in participants who used a console as their preferred gaming platform than another platform (AOR = 13.031, *p*-value = 0.010, CI = 1.866–91.015). Meanwhile, prolonged gaming time of more than four hours a day led to an increased risk of developing IGD up to nine times compared with lesser playing time (AOR = 8.929, *p*-value = 0.011, CI = 1.659–48.050). Those with higher DASS-21 scores for the stress domain had 13 times more risk of developing IGD (AOR = 13.729, *p*-value = 0.001, CI = 2.807–67.136).

## 4. Discussion

In this study, it was found that the prevalence of IGD during the COVID-19 pandemic was 9.86%. The prevalence recorded was higher compared with the worldwide prevalence of 3.05% and several other developing countries, including Sri Lanka (5.06%), Mexico (5.2%), and Indonesia (2.03%) [34,35,36]. The high prevalence number could be attributed to several factors. For instance, the Movement Control Order imposed by the government to control the COVID-19 pandemic has caused people to spend more time on their devices for work and entertainment purposes. In addition, advancements in gaming technology have contributed to the high prevalence of IGD. Stable internet connectivity, wide accessibility, and cheaper devices were among the factors that made online games the preferred activity compared with other activities that could only be performed outdoors and required more money [9].

Furthermore, it was discovered that males have a higher tendency for developing IGD compared with females, which is in line with several other studies stating that males are highly associated with internet gaming addiction problems [7,34]. Despite having a lower number of IGD cases in men for this study, males have a higher risk (AOR = 3.426) to develop IGD in comparison to females. Biologically, male gamers were reported to have a stronger craving for game stimuli and a higher tendency to spend more time playing games [37]. In addition, most of the gaming content that used violence and sexuality elements made the games more appealing to men [38]. This factor made men more susceptible to developing IGD compared with females. However, past research focusing on the relationship between sex and IGD presented that females were vulnerable to IGD as well [39].

Despite smartphones and tablets being the most used devices for playing games, gamers who utilise gaming consoles as their main gaming platform showed a higher tendency to develop IGD. The latest gaming consoles on the market offer a similar gaming experience compared with playing on other devices, where online platform games can be played with high-quality graphics and immense gameplay. Plus, the development of hybrid consoles, which can be played on home consoles or portable devices such as the Steam Deck and Nintendo Switch, made gaming consoles more addictive. The usage of devices increased significantly during the COVID-19 pandemic due to movement restrictions and limited social interaction. As a result, people tend to spend more time using devices as a medium to occupy their free time [40]. A long duration of gameplay between 15 and 20 h every week is classified as overplay, whereas a duration of gameplay of more than 21 h every week (an average of 3 h every day) will start to have a detrimental impact on well-being and increase the risk of developing IGD [13,14].

Psychological stress is often cited as one of the main factors causing many psychiatric illnesses or aggravating a pre-existing psychiatric condition. The failure of coping mechanisms to control the stress had led to depression, anxiety, and IGD. In this study, participants with high stress levels were associated with a higher risk of developing IGD. This study was conducted during the COVID-19 pandemic, when the majority of participants were affected in all major areas of life, including financial, social, and educational, leading to high levels of psychological stress [28]. The high usage of internet gaming is often used as part of a coping mechanism against ongoing stress. It also acts as a source of escapism for certain people who refuse to deal with their daily life problems [39,40]. Psychological 255 interventions such as cognitive-behavioural therapy (CBT) were widely studied and 256 proven to be effective in reducing stress and helping people with IGD [41].

## 5. Limitations and Suggestions

Several limitations should be considered in this study. Since this study was designed as a cross-sectional, the results can only reflect the situation when the study was carried out. By using a cross-sectional design, the temporal relationship between independent variables and the outcome could not be established. Plus, despite the focus given to many factors that influence the development of IGD, many confounding factors that increase the risk of IGD were neglected, such as gaming genre and social support. Thus, future research using a cohort study design with more confounding factors should be explored further.

The second limitation involved the study sample that was taken from a university setting, with the majority of the sample involving young adults. Using the sample from the same setting led to generalisation and did not reflect the real prevalence rate of IGD, especially when the pattern nowadays showed the number of IGD being significant among adolescents and children as well [42]. Another limitation related to the selection of the sample included the distribution of races involved in the study, where almost 80% of the respondents were Malay. This was due to the distribution of students in each of the universities, where the majority of the students are Malay. Thus, large-scale survey research that is more inclusive with the involvement of several other institutions will be needed in future studies to assess the prevalence rate of IGD.

Another limitation was the presence of measurement errors. The data were obtained using three different sets of self-rated questionnaires online. Despite the questionnaires being distributed randomly, some of the participants were hesitant to answer some of the questions truthfully, as some of the questions were sensitive, such as the usage of illicit substances. Some of the participants might also have difficulty justifying the level of severity of the symptoms they experienced, making their responses inaccurate. The implementation of a face-to-face interview is recommended for future research to improve data accuracy and the participant’s understanding should they encounter any problems.

### Recommendations

Further studies involving a larger sample size with diverse socio-demographic backgrounds are recommended to evaluate the real prevalence rate of IGD. Future studies are also recommended to include other covariates, such as preferred gaming genre, social support history, and illicit substance addiction issues, as factors influencing the development of IGD.

## 6. Conclusions

IGD is an emerging psychiatric illness that needs to be taken more seriously. Based on the number of studies in the past, the prevalence rate showed a significant increment, especially in developed and developing countries, during the pandemic period compared with the rates found before the pandemic period. Sex, type of gaming platform, extensive duration of game playing time, and stress were among the factors found to be significantly correlated with the development of IGD. This present study suggests the need to control internet gaming overuse by implementing good gaming hygiene that involves monitoring gaming duration and promoting a healthy lifestyle. This present study also indicates the need for stress and coping skills management targeted at university students to prevent the occurrence of IGD.

## Figures and Tables

**Table 1:** Socio-demographic profile of participants (*n* = 213).

Variables	*n*	%	Mean	Std. Dev.
Sex Male Female	117 96	54.9 45.1		
Race Malay Chinese Indian Others	177 12 19 5	83.1 5.6 8.9 2.3		
Age			21.37	2.292
Preferred Gaming Platform Computer/Laptop Handphone/Tablet Console—PlayStation/Xbox/ Nintendo Switch	37 164 12	17.4 77.0 5.6		
Duration of Playing Time Less than 2 h Between 2 and 4 h More than 4 h	76 71 66	35.7 33.3 31.0		
More Playing Time During Pandemic Yes No	195 18	91.5 8.5		
Substance Use Yes No	15 198	7 93		

**Table 2:** Association between sociodemographic profile, gaming elements, history of substance use, depression, anxiety, and stress with IGD (*n* = 213).

Variable	IGD		Statistical Test		
	Yes *n* (%)	No *n* (%)	*X^2^*	df	*p*-Value
Sex Male Female	6 (2.8) 15 (7)	111 (52.1) 81 (38)	6.538	1	0.011 *
Race Malay Chinese Indian Others	14 (6.6) 3 (1.4) 3 (1.4) 1 (0.5)	163 (76.5) 9 (4.2) 16 (7.5) 4 (1.9)	5.183	3	0.159
Preferred Gaming Platform Computer/Laptop Handphone/Tablet Console—PlayStation/Xbox/Nintendo Switch	3 (1.4) 13 (6.1) 5 (2.3)	34 (16) 151 (70.9) 7 (3.3)	14.478	2	<0.001 *
Duration of Playing Time Less than 2 h Between 2 and 4 h More than 4 h	2 (0.9) 6 (2.8) 13 (6.1)	74 (34.7) 65 (30.5) 53 (24.9)	11.813	2	0.030 *
More Playing Time During Pandemic Yes No	20 (9.4) 1 (0.5)	175 (89.7) 17 (8.0)	0.410	1	0.522
Substance Use Yes No	6 (2.8) 15 (7.0)	9 (4.2) 183 (85.9)	16.495	1	<0.001 *
Depression Yes No	15 (7.0) 6 (2.8)	99 (46.5) 93 (43.7)	3.003	1	0.083
Anxiety Yes No	18 (8.5) 3 (1.4)	116 (54.5) 76 (35.7)	5.192	1	0.023 *
Stress Yes No	19 (8.9) 2 (0.9)	87 (40.8) 105 (49.3)	15.445	1	<0.001 *

* Significant at *p* < 0.05; IGD, Internet Gaming Disorder.

**Table 3:** Binary logistic regression between sociodemographic profile, duration of gaming time, history of substance use, depression, anxiety, and stress with IGD (*n* = 213).

Variable	OR	AOR	95% CI	*p*-Value
Sex Male Female	1.231 1	3.426	1.274–9.212	0.015
Preferred Gaming Platform Computer/Laptop Handphone/Tablet Console—PlayStation/Xbox/Nintendo Switch	1 1.013 2.567	2.754 13.031	0.645–11.769 1.866–91.015	0.172 0.010 *
Duration of Playing Time Less Than 2 h Between 2 and 4 h More than 4 h	1 1.735 2.189	5.670 8.929	0.949–33.887 1.659–48.050	0.057 0.011 *
Stress Yes No	2.619 1	13.729	2.807–67.136	0.001 *

* Significant at *p* < 0.05.

## Data Availability

The data presented in this study are available upon request from the authors.
